# Complete Genome Sequences of Two Shiga Toxin-Producing *Escherichia coli* Strains from Serotypes O119:H4 and O165:H25

**DOI:** 10.1128/genomeA.01496-15

**Published:** 2015-12-17

**Authors:** Rebecca L. Lindsey, Kristen Knipe, Lori Rowe, Lisley Garcia-Toledo, Vladimir Loparev, Phalasy Juieng, Eija Trees, Nancy Strockbine, Devon Stripling, Peter Gerner-Smidt

**Affiliations:** Centers for Disease Control and Prevention, Atlanta, Georgia, USA

## Abstract

Shiga toxin-producing *Escherichia coli* (STEC) is an important foodborne pathogen. Here, we report complete whole-genome sequences for two STEC strains of serotypes O119:H4 and O165:H25 isolated from clinical cases in the United States.

## GENOME ANNOUNCEMENT

Shiga toxin-producing *Escherichia coli* (STEC) is a common cause of foodborne disease (1) that can cause severe illness in the form of bloody diarrhea and hemolytic-uremic syndrome (HUS). There are very few reference sequences available for non-O157 STEC serogroups outside the “big six” that are regarded as adulterants by the USDA. O119 and O165 are both relatively common diarrheagenic serogroups, and serogroup O165 has also been associated with an outbreak in Oregon in 2006 (2, 3). Here, we report the availability of closed whole-genome sequences for O119:H4 and O165:H25 STEC. These closed genome sequences represent STEC serotypes that did not previously have publicly available high-quality reference sequences generated by PacBio sequencing and confirmed with optical mapping.

*E. coli* genomic DNA was extracted according to the manufacturer’s protocol (ArchivePure; 5 Prime, Gaithersburg, MD). DNAs were sheared to 10 kbp or 20 kbp utilizing g-Tubes (Covaris, Inc., Woburn, MA). The sheared products were further size selected utilizing 0.45× AMPure beads. Sheared DNAs were used to generate large SMRTbell libraries using the standard library protocols of the Pacific Biosciences DNA template preparation kit (Menlo Park, CA). The finished libraries were bound to proprietary P5 polymerase and sequenced on a PacBio RSII sequencer using C3 chemistry for 150-min movies. Sequence reads were filtered and assembled *de novo* utilizing the PacBio Hierarchical Genome Assembly Process version 3 (4). The resulting chromosome assemblies were confirmed using OpGen (Gaithersburg, MD) whole-genome maps (WGM). WGM were generated according to the OpGen protocol. Isolate number 2009C-3133 (O119:H4, *stx*_1_ positive, 10-kb library), isolated from a patient in New York in 2009 (accession no. CP013025), was assembled into one closed chromosome with a length of 5,155,368 bp and a G+C content of 50.76%. There are three unnamed plasmids associated with 2009C-3133: accession no. CP013024 is 120,611 bp (closed), accession no. CP013026 is 63,800 bp (closed), and accession no. CP013027 is 174,564 bp. Isolate number 2012C-4227 (O165:H25, *stx*_1_ and *stx*_2_ positive, 20-kb library), isolated from a patient with diarrhea in Oregon in 2009 (accession no. CP013029), assembled into one closed chromosome with a length of 5,202,850 bp and a G+C content of 50.67%. There are two unnamed plasmids associated with 2012C-4227: accession no. CP013028 is 74,656 bp (closed), and accession no. CP013030 is 97,704 bp (closed). The sequences were annotated with the NCBI Prokaryotic Genomes Automatic Annotation Pipeline (5).

A detailed report on an additional analysis of the draft genome sequences will be included in a future publication.

### Nucleotide sequence accession numbers.

The annotated whole-genome *E. coli* sequences have been deposited in DDBJ/ENA/GenBank under the accession numbers CP013024 to CP013030. The versions described in this paper are the first versions.
